# Zinc Finger Nucleases as tools to understand and treat human diseases

**DOI:** 10.1186/1741-7015-8-42

**Published:** 2010-07-01

**Authors:** David Davis, David Stokoe

**Affiliations:** 1 Department of Molecular Biology, Genentech Inc, 1 DNA Way, South San Francisco, California 94080, USA

## Abstract

Recent work has shown that it is possible to target regulatory elements to DNA sequences of an investigator's choosing, increasing the armamentarium for probing gene function. In this review, we discuss the development and use of designer zinc finger proteins (ZFPs) as sequence specific tools. While the main focus of this review is to discuss the attachment of the FokI nuclease to ZFPs and the ability of the resulting fusion protein (termed zinc finger nucleases (ZFNs)) to genomically manipulate a gene of interest, we will also cover the utility of other functional domains, such as transcriptional activators and repressors, and highlight how these are being used as discovery and therapeutic tools.

## Introduction

The expression repertoire of all the genes in any given cell is governed by transcriptional activators and repressors that bind to specific sites in the genome. There are several protein folds that can elicit sequence specific DNA binding, including helix-turn-helix, leucine zipper and zinc finger domains. The C2H2 zinc finger motif, which comprises 20 to 30 amino acids containing two Cys and two His residues coordinated by a zinc atom [1], has proven to be particularly versatile for protein engineering applications. An archetypal member of this family is Zif268, also known as EGR1, which is a transcriptional regulator initially found in mice. The crystal structure of the mouse Zif268 three zinc finger peptide bound to its target DNA sequence showed that the individual zinc fingers fold into two antiparallel β sheets and an α helix, with the α helix making sequence specific DNA contacts in the major grove of the DNA [2]. Each zinc finger binds three nucleotides, with the entire Zif268 polypeptide binding a nine base pair (bp) GCG-TGG-GCG DNA motif. Such a modular design immediately suggested the possibility for combining zinc fingers with distinct triplet recognition motifs, to create proteins that could potentially recognize any DNA sequence. This could include pre-existing zinc fingers with known triplet binding sequences, as well as entirely novel *designer *zinc fingers generated against new DNA sequences [3,4]. Indeed, even before the crystal structure and mode of binding was known, systematic mutations in the α helix of the second zinc finger of the Sp1 transcription factor had been shown to shift DNA binding specificity [5]. Moreover, *mix and match *shuffling of individual zinc fingers was shown to shift DNA binding specificity towards contiguous triplet DNA sequences accordingly [6].

### Methods for design, testing and implementation of zinc finger proteins (ZFPs)

**Figure 1:**
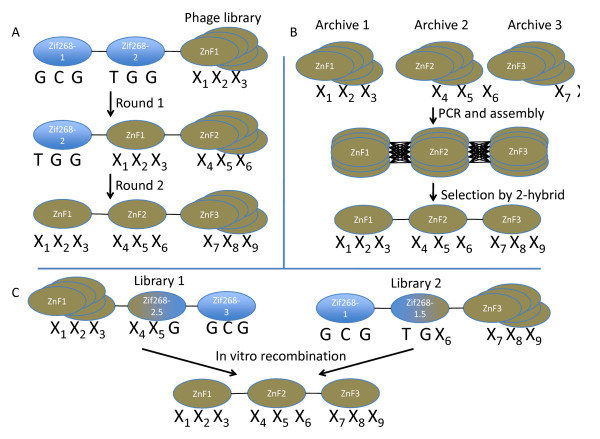
**Strategies for generation of designer zinc-finger proteins**. **A**. Greisman *et al*. [12] described the process of *growing *an artificial zinc-finger through the sequential panning of a phage library for each of the three fingers. **B**. OPEN zinc finger design selects zinc-fingers by screening an archive of characterized zinc fingers for each DNA triplet, followed by random PCR assembly and selection of the best assembled zinc finger trimer. **C**. The bipartite design relies on two libraries in which either the first half (library 1) or the second half (library 2) of the Zif268 zinc finger DNA binding residues has been randomized.

Since the initial proof of concept studies, the design of highly functional ZFPs has made several significant advancements. The first generation ZFP design entailed the use of a *modular assembly*, in which individual zinc fingers were optimized against target triplet DNA sequences, and linked together to form three- or four-ZFPs against 9 or 12 bp sequences. While modular assembly provided examples of successfully applied ZFPs, it was observed that a high failure rate occurred with this approach [7]. Because this deficiency is likely due to the influence that neighboring zinc-fingers have on the sequence specificity of a given zinc finger [8-11], selection-guided assemblies were developed [12-17]. For example, Greisman *et al *described a method to *grow *zinc finger modules, using the first two zinc fingers of the Zif268 trimer against its 6 bp target to anchor a third zinc finger selected from a phage library using a new 3 bp sequence as *bait*. This process was then repeated two additional times to replace each of the two remaining Zif268 zinc fingers [12] (Figure 1A). The resulting synthetic zinc finger trimer can display high affinity and specificity towards the desired 9 bp target sequence. A similar concept was employed by Joung *et al*., who used a bacterial two hybrid system to optimize zinc finger binding to DNA sequences of interest [17].

One of the more advanced selection-based design strategies currently in use is referred to as the OPEN (Oligomerized Pool Engineering) design [18,19]. This approach is an outcome of the Zinc Finger Consortium, a collaboration across multiple academic labs interested in promoting the application of ZFPs as a research tool. As described in detail by Maeder *et al*. [18], OPEN relies on an archive of pre-characterized zinc-finger pools that are organized based on their binding specificity to a given 3 bp sequence. After identifying the desired genomic target sequence, the appropriate mixtures of zinc-finger pools are then randomly assembled using overlapping PCR and screened for the zinc finger assembly displaying the most potent binding activity to the desired 9 bp target (Figure 1B). A temporary caveat of the OPEN design strategy is that the archived pools of zinc-fingers do not yet contain all the possible triplet subsites for a 9 bp target sequence. Currently, only zinc finger pools for all 16 possible GNN subsites (where G is a guanine and N is any base), and a subset of the TNN (where T is a thymine and N is any base) subsites have been identified [18]. Completing the zinc finger library for the remaining possible sequence variables will clearly increase the ability to design ZFPs against challenging targets.

Another selection-based approach for zinc finger design utilizes premade bi-partite libraries [14]. The libraries are composed of two zinc finger pools. In library one, all the base-contacting positions of the first zinc finger and some of the second are randomized, while in library two the remaining base-contacting positions of the second zinc finger and all the base-contacting positions of the third are randomized. The remainder of the zinc finger trimer is the Zif268 sequence in each case. These libraries are individually screened against a chimeric DNA containing the Zif268 target DNA sequence fused to the DNA sequence of interest. Finally sets of *one-and-a-half *zinc fingers are fused together to form proteins that recognize the desired 9 bp target sequence (Figure 1C). This approach was initially used to construct three finger ZFPs that bind to sequences within the HIV promoter [14]. Advantages of the bipartite library screening approach include the ability to generate DNA targets beyond the GNN sequence restrictions of earlier methods. In addition, the overlapping randomization of the two libraries allows simultaneous screening, greatly enhancing the speed at which a zinc-finger assembly with the desired sequence specificity is generated.

More recently, extensive collections of one-finger and two-finger subunits with known DNA binding specificities have been mixed and matched to generate individual four-finger, five-finger and six-finger ZFNs [20]. This approach, a further extension of the modular assembly technique, allows the use of ZFPs with longer recognition sequences, which may afford greater specificity and efficacy.

There remains much debate about the best and most efficient method for zinc finger design (recently discussed in [21,22]). While the modular assembly approach is the quickest and least labor-intensive method to assemble potentially active ZFP trimers, the initial time saving may be offset by the number of proteins required to screen for functional designs. In contrast, selection based designs such as the OPEN system involves greater investment up front, but may generate ZFPs with a higher probability of functional activity to their intended target DNA sequences.

Intrinsic to the methods of the above selection-based designs is the ability to rank the affinities of resulting zinc finger trimers. The ability to select zinc fingers with the highest binding affinity has been shown to be important, as this process usually appears to also increase the sequence selectivity [13,16]. Cornu *et al *demonstrated that specificity generally inversely correlates with toxicity [15]. The goal for optimal zinc finger design is to generate high affinity to intended target, with low affinity to additional sites in the genome [16]. Toxicities associated with one class of zinc finger proteins are also discussed below separately.

### Addition of functional domains expands the utility of ZFPs

**Figure 2:**
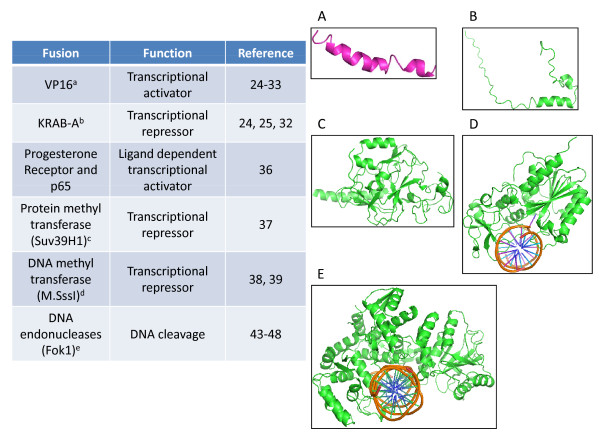
**Functional domains attached to zinc finger proteins**. This figure presents a summary of the functional domains that have been demonstrated to be targeted to specific DNA regions by zinc finger proteins. Shown are the structures of the VP16 transactivation domain from the complex with PC4 (2PHE), the Kruppel associated box domain (1V65), the histone H3K9 methyl transferase Suv39H1 (2R3A), HhaI DNA methyl transferase (1MHT) and FokI endonuclease (1FOK).

Designer zinc fingers have been shown to be useful by multiple approaches. In the simplest application, Choo *et al *showed that an artificial three-zinc finger polypeptide designed against the genomic sequence surrounding the breakpoint of the oncogenic p190 bcr-abl chromosomal fusion could decrease RNA levels of the bcr-abl fusion protein, as well as decrease the viability of cells reliant on this protein for survival [23]. Excitingly, the utility of ZFPs can be further expanded through the addition of functional domains such as transcriptional activation or repression domains (summarized in Figure 2). For example, a sequence-specific transcriptional activator was generated by fusing two three-finger cassettes to the Herpes Simplex Virus transcriptional activator protein VP16 [24]. This was shown to be a functional transcriptional activator when the 18 bp target sequence was incorporated into a reporter plasmid. Conversely, fusion of the six-finger protein to the minimal human Kruppel-associated box-A (KRAB-A) repression domain resulted in a strong decrease in reporter gene expression [24]. This concept was extended to control endogenous genes. For example, Beerli *et al*. [25] fused a six-finger ZFP that recognizes a unique 18 bp sequence in the 5' untranslated region of the proto-oncogene Her2 to either the KRAB-A repression or the VP16 transactivation domains. These proteins decreased or increased endogenous Her2 expression respectively [25]. The authors further applied this concept to Her3, targeting an 18 bp motif in the Her3 5' UTR that only differed by three bases from the Her2 sequence. Remarkably, the Her3 specific zinc finger chimeras only affected the expression of Her3, and not Her2, and vice versa. By placing a tetracycline responsive element upstream of the zinc finger chimeras and expressing in cells together with the tetracycline controlled transactivator, the expression levels of Her2 could be placed under the control of doxycycline [25]. Moreover, the decreased levels of Her2 were shown to have functional consequences, causing cell cycle arrest. Zhang *et al *[26] expanded this work, by designing 10 independent ZFPs fused to the VP16 transactivation domain that recognize 9 bp sequences within -70 to -860 bps from the transcriptional start site of the erythropoietin promoter. This group made the important observation that DNA binding affinity is not strictly related to the ability to stimulate transcription, although high affinity interactions are required to see functional effects. Therefore, chromosomal DNA structure and organization is likely to restrict access to novel designer zinc fingers.

Additional endogenous genes that have been successfully targeted by ZFPs linked to transcriptional activation domains include *VEGF-A *[27], *PPARγ1 *and *PPARγ2 *[28], *bax *[29] and *fushi tarazu *(drosophila) [30]. Even the expression of transgenes driven by strong viral promoters can be increased using this approach, as demonstrated by Reik *et al*. [31]. In this study the authors used DNAseI to identify accessible regions of chromatin within the SV40 promoter, and generated three-finger ZFPs recognizing these sites. Using this method, they demonstrated a two-fold increase in the expression of an IgG transgene driven by the SV40 transgene in CHO cells [31], an approach that could have significant use for increasing the industrial production of therapeutically relevant proteins and antibodies. A more comprehensive review of the use of ZFPs to increase or decrease transcription of target genes can be found in Jamieson *et al*. [32]. Gene induction using ZFPs has even been extended to *in vivo *use in mice, by infecting mouse ears with adenoviruses containing ZFP-VP16 fusions targeted to the VEGF-A promoter, resulting in increased local angiogenesis [33]. Potential therapeutic uses of ZFPs causing increased VEGF levels are discussed further in [34].

Some recent advances in the use of inducible systems to regulate endogenous gene expression using ZFPs have also been documented. For example, Pollock *et al*. split the ZFP DNA binding and VP16 transcriptional activation domains into two polypeptides separated by an internal ribosomal entry site (IRES), each fused to either 3xFKBP or mutant FRB domains, using the synthetic rapamycin derivative AP21967 to induce dimerization. This approach allowed inducible regulation of VEGF-A expression from its endogenous locus [35]. Another approach involved generation of a chimeric protein consisting of a ZFP targeted against the VEGF-A promoter, the ligand binding domain of the progesterone receptor, and the p65 NFκB transactivation domain. In the absence of the progesterone receptor agonist mifepristone, the ZFP-PR-p65 fusion protein was bound to heat shock proteins in cytosol and was unable to regulate transcription. Mifepristone addition resulted in dissociation of HSPs, nuclear translocation and transcriptional activation of VEGF-A [36].

Finally, in addition to transcriptional activators and repressors, other regulatory domains can be fused to ZFPs, allowing additional control of endogenous gene expression. For example, Snowden *et al*. [37] fused the Histone Methyl Transferase Domains of G9A or Suv39H1 to a previously described [27] ZFP targeted to the VEGF-A promoter. These proteins decreased endogenous VEGF-A expression, which was associated with increased lysine-9 methylation of the Histone H3 associated with the VEGF-A promoter. Repression could be further stimulated by co-expression of an additional ZFP fused to the v-erbA domain that recruits Histone Deacetylase complexes. Finally, ZFP fusions to DNA methylases have also been reported, both as an intact methyl transferase fused to one three-finger ZFP [38], and as two independent ZFPs fused to methyl transferase fragments that self-assemble upon colocalization at the correct DNA motif [39]. While promising, this particular application has not been demonstrated to apply to single site modification of complex genomes. Nevertheless, these experiments demonstrate that ZFPs represent powerful tools for precise modification of chromatin with subsequent control of local gene expression.

### Addition of endonuclease activities to ZFPs to create targeted DNA scissors

**Figure 3:**
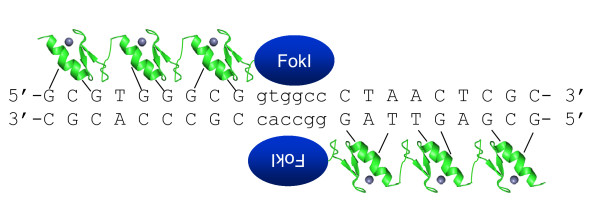
**Site specific cutting requires heterodimerization of two independently designed ZFNs**. Each ZFN binds to at least nine nucleotides and flanks a 5 to 6 bp spacer.

A further breakthrough in the utility of designer zinc finger proteins has been the ability to cleave specific sites in large genomes. The initial proof-of-concept experiment in this area was the demonstration that the FokI endonuclease from the prokaryote *Flavobacterium okeanokoites*, can be separated into two functional domains - one that binds DNA in a sequence specific manner, and one that cleaves in a sequence independent manner. Moreover, the cleavage domain can be fused to unrelated DNA recognition modules and retain DNA cleavage, which occurs at 9 and 13 bps from the recognition site [40]. Structural and experimental analysis have shown that the nuclease domain of FokI requires dimerization for endonuclease activity on the target DNA [41,42]. This fact conveniently increases the potential specificity for the use of ZFPs linked to the FokI endonuclease (hereafter termed zinc finger nucleases, or ZFNs). Therefore by fusing a FokI monomer to two ZFPs, which bind to adjacent sequences, will generate at least an 18 bp sequence specific DNA nuclease that in principle should allow selective targeting within mammalian genomes. This approach is depicted in Figure 3, in which two zinc finger trimers are bound to their cognate DNA sequences creating an active FokI dimer. The effectiveness of this strategy was initially shown with artificial DNA substrates *in vitro *[43] and integrated transgenes in *Xenopus *eggs [44]. Subsequently, advances in the design of ZFPs led to the successful applications of this technique to endogenous genomic loci in drosophila [45], human cells [46], plants [47] and *C. elegans *[48]. The remainder of this review will focus on the consequences and applications of targeted single stranded breaks induced by such ZFNs.

### Targeted mutations induced by ZFN cleavage

The consequences of a double strand break induced by targeted ZFN expression vary upon several different factors, but in general cells repair these breaks by either non-homologous end joining (NHEJ) or homologous recombination (HR) (recently reviewed in [49]). The FokI-mediated DNA cleavage leaves overhanging ends, which can either be filled in, completely or incompletely, or chewed back by limited exonuclease activity, followed by ligation by endogenous DNA ligase activity. In some cases small regions of single stranded microhomology are used to initiate strand annealing and ligation. The net result of this process is that small insertions or deletions are usually generated in the region flanking the ZFN-mediated processing. When the ZFPs are targeted to coding regions, the outcome is often a shift in the reading frame, usually leading to a null allele of the targeted gene. Examples of the consequences of ZFN-mediated cleavage and repair are described in references [50] (drosophila) and [51] (human). When applied to mammalian cells, this approach presents an attractive alternative to previous labor- and time-intensive techniques using homologous recombination and gene targeting (reviewed in [52]). Although this has only been tested for a relatively small number of genes in mammalian cells so far, the results seem encouraging. An initial proof of concept study was performed using the dihydrofolate reductase gene (*DHFR*) in Chinese hamster ovary (CHO) cells [53]. Using two distinct pairs of ZFNs that show effective targeting of exon 1 of the *DHFR *gene, CHO cells were transfected with these ZFNs and cloned by limiting dilution. For the two ZFN pairs in two experiments, 5/68 (7%) and 11/350 (3%) of single cell clones showed alteration of the target *DHFR *genes. Culture conditions, the sequence of the zinc fingers, and the nature of the FokI enzymes likely contributed to the difference in targeting efficiency between the two experiments. However, in both experiments approximately 30% of the clones showing *DHFR *disruption expressed no wild type (WT) allele, and both alleles were shown to be independently mutated. Mutations at the *DHFR *loci were consistent with targeted FokI cleavage and displayed phenotypic effects expected from cells lacking functional *DHFR *[53].

To target a more therapeutically relevant gene, Perez *et al*. generated a pair of ZFNs targeted to the first coding exon of *CCR5*, the major co-receptor for HIV entry [51]. Using either adenovirus or electroporation, these ZFNs were introduced into human T cell lines, resulting in decreased expression of *CCR5*, as well as protection from HIV infection. Sequencing clones that proliferated following infection with HIV showed the expected disruption of the endogenous *CCR5 *gene, with insertions and deletions of between 1 and 43 nucleotides following cleavage and repair. Single cell cloning of 52 individual infected primary T cells showed that 12 (23%) contained a modified *CCR5 *allele; of these, four (33%) clones showed bi-allelic modification. This approach was extended to a mouse model for HIV infection, where expansion of the *CCR5 *modified cells was observed and corresponded with lower levels of viremia [51]. Based on these exciting preclinical studies, *CCR5 *directed ZFNs are now in phase I human trials to explore their therapeutic value against HIV.

Since these recent reports, additional genes have been knocked out in mammalian cells using this technique with similar frequencies and efficiencies. As many as three genes have been biallelically disrupted in a single cell line using these reagents either sequentially, or simultaneously [54]. These results show the generality of these methods, as well as their potential for multi-gene analyses.

### Gene replacement induced by ZFNs

The second method used by cells to repair damaged DNA is homologous recombination (HR). This can be favorably exploited in the context of ZFN-mediated cleavage by introducing exogenously added donor plasmids containing homologous DNA stretches. Initial proof-of-concept experiments in human cells showed the feasibility of this approach by demonstrating the ability to repair a mutated form of GFP stably integrated into the genome [46]. The first example of ZFN-mediated repair of an endogenous mutated human gene was presented by Urnov *et al*. [55]. In this study, the authors designed ZFNs targeting *IL2Rγ*, the gene mutated in X-linked severe combined immune deficiency (SCID). They used ZFNs targeted to exon 5 of *IL2Rγ*, and a plasmid carrying this exon with a restriction site to monitor incorporation, flanked by approximately 1 kb homology either side of the mutation. Co-transfection of ZFNs and this plasmid into K562 cells showed a 10 to 20% modification rate of the *IL2Rγ *exon 5 across a polyclonal cell population, with similar frequencies documented by limiting dilution cloning (13/96 (14%) heterozygous clones, of which 6/9 (67%) were homozygous for the introduced mutation). The authors then introduced a frameshift mutation into one or both alleles of *IL2Rγ *using these reagents, thereby mimicking the defect seen in the human disease, and finally fixed the mutation using a donor plasmid containing the wildtype exon. This latter experiment shows the path for use of this approach in gene therapy. Primary patient T cells could be extracted and modified *ex vivo*, before subsequent re-implantation. A huge benefit of this approach compared to viral based gene therapy methods, is the elimination of potential detrimental gene reactivation events due to insertional mutagenesis [56].

Mouse cells have also been shown to be responsive to ZFN-mediated genome editing through homologous recombination. Melanocytes derived from albino mice contain a point mutation in the first exon of the tyrosinase gene. Cotransfection of these melanocytes with ZFNs targeting a sequence 80 bps from the *Cys85Ser *mutation, together with the WT donor sequence resulted in pigmented cells as early as four days post-transfection [57]. Finally, successful genetic modification of mouse ES cells has also been enabled by ZFNs, which is discussed in more detail in a later section.

### Toxicities associated with ZFNs

While a pair of three-finger ZFNs targeting an 18 bp sequence should in theory generate a specificity of 7 × 10^10 ^(greater than the size of the human genome), in practice more than one site often appears to be cleaved under these conditions. This is due to the ability of these zinc fingers to recognize one or two bp variants of the predicted site, as well as the ability of earlier ZFNs to function as both homo and heterodimers. As these reagents are currently being used in clinical trials, it is important to understand in detail the cause, extent and consequences of off-target toxicities. Several approaches have been used to determine the *off-target *frequency of DNA cleavage and subsequent toxicity. One of these approaches simply monitors the presence of the transfected cells over time, looking for cell-intrinsic toxicity leading to the disappearance of the transfectants. Using this assay, it was observed that, in general, ZFNs with a higher affinity to their target site were less toxic than ZFNs targeting the same site but with lower affinity [15,16]. The expression levels of ZFNs have also been shown to correlate with the degree of apoptosis, as measured by the presence of sub-G1 DNA content [58], while the use of poorly designed ZFNs have been shown to increase the level of γH2AX or p53BP1 expression as compared to optimally designed ZFNs targeting the same site [16].

Another method for assessing possible off-target cutting is to identify genomic sites that are very similar, but not identical, to the predicted cutting site. In the study by Perez *et al*. using four-finger ZFNs targeted to *CCR5*, it was noted that the same region in *CCR2 *differed by only 2 bps, one per 12 bp zinc finger recognition motif. Detection of modification at this site, either by a Cel-I nuclease assay that measures DNA mismatches, or by direct sequencing of multiple targeted clones, showed a 10-fold decreased activity at this site relative to *CCR5 *[51]. An additional 13 genomic loci that differed from the *CCR5 *target sequences by 3 bps or less were also identified. Deep sequencing of these regions following expression of the *CCR5 *ZFNs showed only very rare modification (2/38,023 sequences examined) at one locus, an intron of the gene *ABLIM2*. Therefore, this study shows that optimally designed ZFNs can exhibit very limited off-target cutting.

A recent advance in the design of ZFNs appears to have significantly decreased the incidence of off-target endonuclease activity. In the original design, either zinc finger components of the intact ZFN pair could self dimerize to form the active FokI nuclease, meaning that three putative genomic sites could be perfect match targets for the transfected ZFNs rather than just one. In addition, any 1 or 2 bp tolerance with either zinc finger also dramatically increases the number of potential off-target sites due to homodimerization (discussed in [59]). Analysis of the FokI dimer structure identified residues that form the interface, and suggested mutations that could prevent homodimerization and therefore allow only heterodimerization. Miller *et al*. [60] showed that mutation of two amino acids in each dimer partner (E490K/I538K and Q486E/I499L) dramatically reduces activity as homodimers, but retains the majority of its activity as a heterodimer. A similar approach by Szczepek *et al*. [61] also identified pairs of FokI mutants that preferentially acted as heterodimers. As predicted, these FokI heterodimerization mutants show reduced off-target cutting, as measured by γH2AX and p53BP1 foci [60,61].

Additional reported modifications to the ZFN reagents to reduce toxicity and off-target cutting include destabilizing the ZFN proteins themselves. This follows from the observation that high levels expression of ZFNs are toxic [16,58]. Attaching ubiquitin to the N-terminus of ZFNs was shown to destabilize these proteins, which could be overcome by the addition of the proteasome inhibitor MG132. A second approach utilized fusions to a FK506 Binding Protein (FKBP) domain that is rapidly degraded unless a rapamycin analogue was added. The authors propose that these methods generate a higher gene targeting to toxicity ratio, using viability and p53BP1 staining as readouts [62].

### Application of ZFNs to model organisms

A significant advantage of ZFNs is the ability to genetically manipulate previously intractable model organisms. Selective genetic modification has been demonstrated with the injection of ZFNs into embryos of *C. elegans *[48], zebrafish [63-65], drosophila [66] and rat [67]. The successful application of ZFNs to selectively target a given gene in these organisms greatly expands their potential utility and value as models for human disease. For example, the rat has long been understood to serve as a valuable model for toxicology and metabolism studies. However, genetic manipulation of this species has been hampered by the lack of pluri-potent, germline competent embryonic stem (ES) cells that can be easily manipulated *in vitro*. The ability of ZFNs to bypass the requirement of ES cells (by direct injection into a single cell oocyte) has allowed the targeted, heritable mono and biallelic disruption of a GFP transgene and three endogenous genes (*IgM*, *Rab38 *and *IL2Rγ*) [67,68]. Although it remains to be demonstrated whether targeted knock-ins can also be achieved with this approach, selective genetic deletions will greatly enhance the value of the rat model. Even for mouse genomic engineering, ZFNs have several advantages over traditional homologous recombination approaches: 1. knockouts can be performed in genetic backgrounds for which ES lines are not available. 2. ZFN microinjection into oocytes decreases the time to generate simple knockouts. 3. ZFNs allow the ability to knockout a gene in already complex genetic backgrounds where other mutations have already been established.

In addition to expanding the value of the above model organisms for human disease research, these models can also enlighten the mechanism of ZFN-derived genetic alterations. A recent study using drosophila demonstrated that DNA repair pathways act on the ZFN induced double-stranded DNA cuts. Moreover, the researchers illustrated the ability to bias the outcome of ZFN cuts towards gene replacement (HR) or NHEJ by manipulating the genes required for each repair pathway [50]. The rapid rate of successful ZFN application to numerous organisms, some previously intractable to genomic manipulation, is quite amazing and suggests this method of genetic manipulation will soon become a standard research tool across many disciplines.

### Novel uses of ZFNs for future therapeutic applications

Although ZFPs and ZFNs are still a recent area of research, there are some immediately applicable therapeutic opportunities that have arisen. For example, Sangamo biosciences have developed ZFPs fused to the VP16 transcriptional activation domain targeted to the VEGF-A promoter which is currently in Phase 2 trials for diabetic neuropathy and Amyotrophic Lateral Sclerosis (ALS). This drug, which involves direct injection of DNA encoding the ZFPs into the affected site, requires efficient uptake of the DNA and strong expression in the target tissues. In addition, ZFNs targeted to the HIV coreceptor CCR5 (described above) for the treatment of HIV/AIDS are in Phase 1 Clinical trials. In this approach, patient T cells are extracted and modified to express the mutant CCR5 allele which is resistant to HIV infection. While it is too early to determine the success or failure of this trial, data from a single patient show that the modified T cells remain in the blood several weeks after infusion of the ZFN treated cells http://investor.sangamo.com/releasedetail.cfm?ReleaseID=247037. Positive selection for these cells in the patient means that high efficiency uptake and expression is not critically required. ZFNs targeted to the glucocorticoid receptor introduced into T cells as a therapy for glioblastoma have also initiated Phase 1 clinical trials http://investor.sangamo.com/releasedetail.cfm?ReleaseID=436582.

There are also much earlier stage experiments ongoing using ZFNs for genomic modification that hold great promise for future therapeutic opportunities. In particular, modification of human stem cells using ZFPs and ZFNs could be used to treat many human diseases. Initial experiments in mouse embryonic stem cells showed that ZFPs fused to transcriptional activator or repressor domains could modify the Oct4 locus, and uncovered an unexpected paradoxical role this gene serves in maintaining pluripotency. In these studies, the repressor ZFP was shown to induce the expected differentiation, while the activator ZFP also surprisingly caused some cell differentiation [69]. Use of ZFNs has also been extended to human ES cells [70]. Hockemeyer *et al *applied ZFNs to introduce GFP into the Oct4 locus allowing the fluorescent tagging of cells depending on their differentiation status. In addition, the authors used ZFNs to target the first intron of the *PPP1R12C *gene using homologous recombination. The authors used both the endogenous *PPP1R12C *promoter as well as the phosphoglycerol kinase (PGK) promoter to drive expression of puromycin resistance. Interestingly, the targeting efficiencies in both cases were quite similar (approximately 50%), with a similar degree (approximately 50%) of non-targeted integration in both cases. Targeting of non-expressed genes was also shown by successfully targeting *PITX3*, a gene that is not expressed in hES cells. Gene repair and gene knockout have also been demonstrated in induced pluripotent stem (iPS) cells [71]. Importantly, experiments performed in all of the above studies show that ZFN modified hES cells retain proliferative capacity and pluripotency, opening the door to modification of disease-related genes in affected individuals.

In some diseases, it is not genomic DNA that is altered, but mitochondrial DNA. Neurogenic muscle weakness, ataxia, and retinitis pigmentosa (NARP) and maternally inherited Leigh's syndrome are caused by a single T8993G nucleotide alteration in mitochondrial DNA [72]. A combination of adding a nuclear exclusion sequence and a mitochondrial targeting sequence was shown to be effective in localizing ZFNs to the mitochondria [73]. However, the conventional heterodimeric ZFNs were unsuccessful at binding and/or cleaving the appropriate site in the mitochondrial genome. Therefore Minczuk *et al *[72] developed a novel single ZFN which expresses two Fok1 proteins separated by a flexible linker, using ZFPs to direct this polypeptide to the T8993G mutation. When expressed in cells expressing copies of both the mutant and WT mitochondrial genome, this *quasi-dimeric *ZFN was able to selectively cleave the mutant copy leading to enrichment of the WT copy, which could be sufficient to reverse phenotypic consequences.

In addition to their use as direct therapeutics, ZFNs can also aid in the drug discovery and/or drug manufacturing process. Chinese hamster ovary (CHO) cells are used to produce many different recombinant proteins for therapy. Cost *et al *[74] describe the use of ZFNs to delete Bax and Bak from CHO cells, making these cells more resistant to apoptosis induced by stresses as a result of growth in large bioreactors. Double knockout cells were obtained by sequentially transfecting CHO cells with BAK ZFNs (2/552 cell clones (0.3%) showed modification of both alleles) followed by BAX ZFNs (21/79 clones (26%) showed deletion of BAX, which is fortuitously hemizygous in this cell line). Double mutant cells were almost completely resistant to apoptosis, and showed a two- to five-fold increase in recombinant antibody production under stressed conditions [74].

Finally, ZFPs and ZFNs can also aid the drug discovery process by creating isogenic lines that vary only by the presence or absence of the drug target of interest. For example Liu *et al*. [75] used ZFPs targeting the parathyroid hormone receptor (PTHR) fused to a transcriptional activation domain, to elevate levels of PTHR in 293 cells which normally do not express this protein, as well as fused to a transcriptional repressor domain to repress expression in SAOS2 cells. Such cell lines create an almost perfect method to discriminate on-target from off-target effects of a given drug.

## Conclusions

Manipulating endogenous genes at will has long been the goal for researchers wishing to understand and treat human diseases. The use of engineered zinc fingers to modify specific genomic loci is a relatively recent addition to this area, but is rapidly showing enormous promise at becoming a reliable research and therapeutic tool. In fact, designer ZFPs are already in clinical trials, and a multitude of others are in development. However, there are a few cautions that prevent unbridled enthusiasm at this time and point to the need for further technical development. For example, it is clear that some regions of the genome are more targetable than others by ZFPs; the reason for this is not completely clear, and until this is resolved, progress will remain somewhat impeded. In addition, the potential toxicities as a result of off-target binding and cutting (in the case of ZFNs) are still not completely understood, and the methods currently available to even monitor these events are laborious. While ZFNs have been successfully employed in numerous model organisms to generate gene knock-outs, the ability of ZFNs to enhance the generation of knock-in animals remains largely untested. Finally, the expansion of therapeutic ZFPs will also be limited by the ability to efficiently deliver the ZFP into the disease relevant cell. Nevertheless, given the explosive progress made in just the last 10 to 15 years, one cannot help but be excited about what will happen in the next equivalent time period.

## Abbreviations

ABLIM2: actin binding LIM protein family member 2; ALS: amyotrophic lateral sclerosis; bp: base pair; C2H2: cysteine2 histidine2; CCR5: C-C chemokine receptor-5; CHO: chinese hamster ovary; DHFR: dihydrofolate reductase; DNA: deoxyribonucleic acid; EGR1: early growth response-1; ES: embryonic stem; FKBP: FK506 binding protein; FRB: FK506 and rapamycin binding; HIV: human immunodeficiency virus; HR: homologous recombination; IL2R: interleukin 2 receptor-; IPS: induced pluripotent stem cells; IRES: internal ribosomal entry site; KRAB-A: Kruppel-associated box-A; NHEJ: non-homologous end joining; OPEN: oligomerized pool engineering; P53BP1: p53 binding protein-1; PGK: phosphoglycerol kinase; PITX3: pituitary homeobox-3; PPAR: peroxisome proliferator nuclear antigen; PTHR: parathyroid hormone receptor; SCID: severe combined immunodeficiency; VEGF: vascular endothelial growth factor; ZFN: zinc finger nuclease; ZFP: zinc finger protein.

## Competing interests

David Stokoe holds a small amount of stock in Sangamo. Genentech and Sangamo have previously collaborated on the use of ZFNs to delete Bax and Bak from antibody producing CHO cells (see reference 74 in the review).

## Authors' contributions

Both authors contributed equally to the writing of this article.

## Pre-publication history

The pre-publication history for this paper can be accessed here:

http://www.biomedcentral.com/1741-7015/8/42/prepub
